# Multi-functionality of a tryptophan residue conserved in substrate-binding groove of GH19 chitinases

**DOI:** 10.1038/s41598-021-81903-3

**Published:** 2021-01-28

**Authors:** Takuya Nagata, Shoko Shinya, Takayuki Ohnuma, Tamo Fukamizo

**Affiliations:** 1 Department of Advanced Bioscience, Kindai University, 3327-204 Nakamachi, Nara, 631-8505 Japan; 2 Agricultural Technology and Innovation Research Institute (ATIRI), Kindai University, Nara, Japan

**Keywords:** Enzyme mechanisms, Glycobiology

## Abstract

GH19 and GH22 glycoside hydrolases belonging to the lysozyme superfamily have a related structure/function. A highly conserved tryptophan residue, Trp103, located in the binding groove of a GH19 chitinase from moss *Bryum coronatum* (BcChi-A) appears to have a function similar to that of well-known Trp62 in GH22 lysozymes. Here, we found that mutation of Trp103 to phenylalanine (W103F) or alanine (W103A) strongly reduced the enzymatic activity of BcChi-A. NMR experiments and the X-ray crystal structure suggested a hydrogen bond between the Trp103 side chain and the -2 sugar. Chitooligosaccharide binding experiments using NMR indicated that the W103F mutation reduced the sugar-binding abilities of nearby amino acid residues (Tyr105/Asn106) in addition to Trp103. This appeared to be derived from enhanced aromatic stacking of Phe103 with Tyr105 induced by disruption of the Trp103 hydrogen bond with the -2 sugar. Since the stacking with Tyr105 was unlikely in W103A, Tyr105/Asn106 of W103A was not so affected as in W103F. However, the W103A mutation appeared to reduce the catalytic potency, resulting in the lowest enzymatic activity in W103A. We concluded that Trp103 does not only interact with the sugar, but also controls other amino acids responsible for substrate binding and catalysis. Trp103 (GH19) and Trp62 (GH22) with such a multi-functionality may be advantageous for enzyme action and conserved in the divergent evolution in the lysozyme superfamily.

## Introduction

Chitin is a linear polysaccharide of *N*-acetylglucosamine (GlcNAc), that is degraded by chitinases (EC 3.2.1.14), which catalyze the hydrolysis of β-1,4-glycosidic linkages of the polysaccharide chains^1^. The enzymes are involved in important biological processes in a wide variety of living organisms, such as self-defense, growth, morphogenesis, and stress tolerance in microbes, plants, insects, fishes, and mammals^2–7^. Most chitinases are classified into two families, GH18 and GH19, according to amino acid sequences (http://www.cazy.org/)^8^. GH18 chitinases are mainly composed of a core TIM barrel fold with a couple of subdomains. They catalyze hydrolysis through a substrate-assisted mechanism with retention of the anomeric form using a catalytic motif, DXDXE, localized inside of the TIM barrel structure^9,10^.

**Figure 1:**
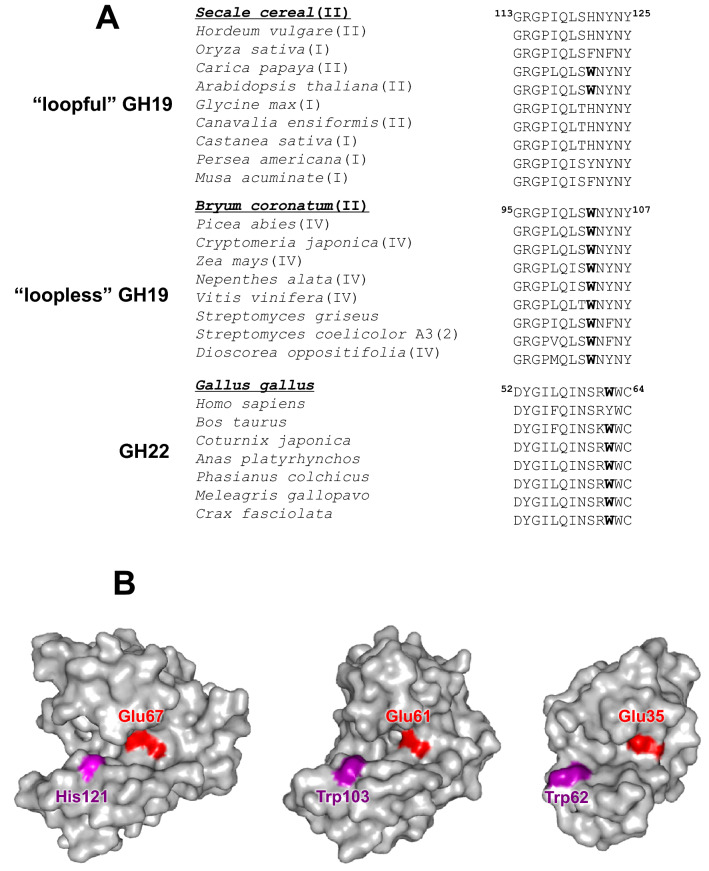
(**A)** Multi-species alignment of amino acid sequences of the β-hairpin region of the GH19 and GH22 glycoside hydrolases. The sequences are from *Secale cereal* (Q9FRV0), *Hordeum vulgare* (AAA56786), *Oryza sativa* (Q7DNA1), *Carica papaya* (P85084), *Arabidopsis thaliana* (AAT41815), *Glycine max* (AAK01734), *Canavalia ensiformis* (CAA07413), *Castanea sativa* (CAA64868), *Persea americana* (CAB01591), *Musa acuminate* (Q8VXF1), *Bryum coronatum* (GenBank Accession No. BAF99002), *Picea abies* (AAQ17051), *Cryptomeria japonica* (BAD77932), *Zea mays* (PWZ36890), *Nepenthes alata* (BBC62322), *Vitis vinifera* (AAQ10093), *Streptomyces griseus* (BAA23739), *Streptomyces coelicolor* A3(2) (BAA75648), *Dioscorea oppositifolia* (BAC56863), *Gallus gallus* (P00698), *Homo sapiens* (CAA32175), *Bos taurus* (AAC37312), *Coturnix japonica* (XP_015711651), *Anas platyrhynchos* (XP_005008937), *Phasianus colchicus* (P00702), *Meleagris gallopavo* (XP_003202118), and *Crax fasciolata* (Q7LZQ3). Species written in bold are the representatives of the individual enzyme families and used for structural display in (**B**). (**B)** Surface models of the crystal structures of “loopful” GH19 chitinase from *Secale cereal* (left, PDB code: 4DWX), “loopless” GH19 BcChi-A (middle, PDB code: 3WH1), and GH22 hen egg white lysozyme (right, PDB code: 1LYZ). The catalytic acids, Glu67 (*S. cereal*), Glu61 (BcChi-A), and Glu35 (lysozyme), are highlighted in red. His121 of the *S. cereal* enzyme corresponds to Trp103 of BcChi-A and is highlighted in purple. Trp103 of BcChi-A highlighted in purple was the mutation target in this study. Trp62 essential for substrate binding in GH22 lysozyme is also highlighted in purple.

In contrast, GH19 chitinases hydrolyze the β-1,4-linkages through a single displacement mechanism with inversion of the anomeric form^11^. As shown in Fig. 1B (left and middle panels), GH19 enzymes are composed of two lobes (upper and lower lobes), both of which are rich in α-helical structures. One group of the GH19 enzymes has six loop structures, which are responsible for substrate-binding at both ends of the substrate-binding groove lying in between the two lobes^12–14^. We designated these GH19 enzymes with six loops as “loopful” chitinases (the left panel of Fig. 1B). The catalytic center is located in the midst of the substrate-binding groove. On the other hand, the other group of GH19 chitinases lack several loops, and are designated as “loopless” chitinases (the middle panel of Fig. 1B). Bacterial GH19 chitinases isolated from *Streptomyces griseus* HUT6037^15^ and *Streptomyces coelicolor* A3(2)^16^, and the GH19 enzyme from the evergreen conifer Norway spruce^17^ are “loopless” enzymes, and their substrate-binding grooves are shorter than those of “loopful” enzymes (Fig. 1B). In fact, the binding groove of a “loopful” GH19 chitinase from rye seeds can accommodate two molecules of *N*-acetylglucosamine tetramer, (GlcNAc)_4_^18,19^, whereas that of a “loopless” chitinase from the moss, *Bryum coronatum* (BcChi-A), accommodates only one (GlcNAc)_4_ molecule^20^.

Lysozymes are self-defense enzymes widely distributed in living organisms, breaking down the cell walls of pathogenic bacteria^21^. The enzymes hydrolyze the β-1,4-glycosidic linkage between *N*-acetylmuramic acid (MurNAc) and *N*-acetyl-d-glucosamine (GlcNAc) of the peptidoglycan chains in bacterial cell walls^22^. Lysozymes also hydrolyze β-1,4-glycosidic linkages of chitin, providing similar functionality to that of chitinases^23^. GH19 and GH22 enzymes represent a lysozyme superfamily together with GH23, GH24, and GH46 enzymes, because they share invariant structural elements, a central α-helix and a β-hairpin, without any sequence similarity^24,25^. Figure 1A shows the amino acid sequence alignment of the β-hairpin region of GH19 chitinases and GH22 lysozymes. In the β-hairpin region, we found a tryptophan residue at the 103rd position (the amino acid number for BcChi-A), which is highly conserved in “loopless” GH19 enzymes (Supplementary Fig. S1). The role of aromatic residues in the binding groove of carbohydrate-related enzymes has been studied intensively by many investigators^26–29^. Especially in tryptophan residues, the indole side chains strongly contribute to the interaction with the pyranose rings, through CH-π stacking and a hydrogen bond. Trp62 in hen egg white lysozyme belonging to the GH22 family is well-known and best characterized with respect to its interaction mechanism, by mutational and chemical modification studies^30–32^. Trp62 is also highly conserved in GH22 lysozymes. Of note, as shown in Fig. 1B, the relative location of Trp103 of BcChi-A is similar to that of Trp62 in hen egg white lysozyme. Although the position of the GH19 tryptophan (Trp103) shifts by two amino acid units to the N-terminal side from the GH22 tryptophan (Trp62), functional similarities may exist between these two tryptophan residues.

In this study, we mutated Trp103 of BcChi-A, and the mutated enzymes were characterized with respect to their enzymatic activities and the chitooligosaccharide binding abilities. NMR spectroscopy was used to analyze the structure and binding ability of the mutated BcChi-A. The experimental data are discussed based on the NMR spectra of wild-type and mutated BcChi-A enzymes and the crystal structure of BcChi-A in complex with (GlcNAc)_4_. The significance of the tryptophan residue in GH19 chitinase is also discussed by comparing with Trp62 in GH22 lysozymes.

## Results

### Production of BcChi-A mutants

The recombinant proteins of the wild-type BcChi-A and the mutants were successfully produced using a pET22b expression system with an *E. coli* strain BL21(DE3)^33^. Purification of the Trp-mutated proteins was also successful using a chromatographic system of Q-Sepharose and Sephacryl S-100 columns. Stable isotope-labeled proteins were also purified successfully, although the yields were lower than those of the unlabeled proteins. Each enzyme protein purified by this system exhibited a single band in SDS-PAGE gels. Yields of the purified unlabeled protein, ^15^N-labeled protein, and ^15^N/^13^C-labeled protein from 1 L of culture medium were 98.1, 40.6, and 22.7 mg for wild-type; 98.3, 37.7, and 18.5 mg for W103F; 106.0, 39.5, and 21.6 mg for W103A; 90.4, 40.0, and 20.5 mg for E61Q; 94.7, 40.2, and 19.5 mg for E61Q/W103F; and 99.5, 37.3, and 21.0 mg for E61Q/W103A, respectively.

### Enzymatic activities of BcChi-A and its mutants

Table 1 summarizes the enzyme activities of wild-type BcChi-A and its mutants. The activity determined based on the reducing sugar release from the substrate glycol chitin decreased to 40% and 13% for W103F and W103A compared with the wild-type, respectively. The other mutants, E61Q, E61Q/W103F, and E61Q/W103A, did not show any activity. When chitopentasaccharide (GlcNAc)_5_ was used as the substrate, W103F and W103A produced equal amounts of (GlcNAc)_2_ and (GlcNAc)_3_ but not GlcNAc and (GlcNAc)_4_ at all, as in the case of wild-type^33^. (GlcNAc)_5_-binding mode was not affected by these mutations. However, the rates of (GlcNAc)_5_ degradation by W103F and W103A were significantly lower than that by wild-type, at 58% and 19%, respectively. Effects of the mutations on the activity toward glycol chitin were more intense than those toward the oligosaccharide substrate.

**Table 1:** Enzymatic activities of BcChi-A and the Trp103-mutated enzymes.

	Glycol chitin		(GlcNAc)_5_	
	Specific activity (U mg^−1^)	Relative activity (%)	Specific activity (μmol min^−1^ mg^−1^)	Relative activity (%)
Wild-type	196.85	100	81	100
W103F	78.74	40	47	58
W103A	25.35	13	15	19

### Thermal unfolding experiments

**Figure 2:**
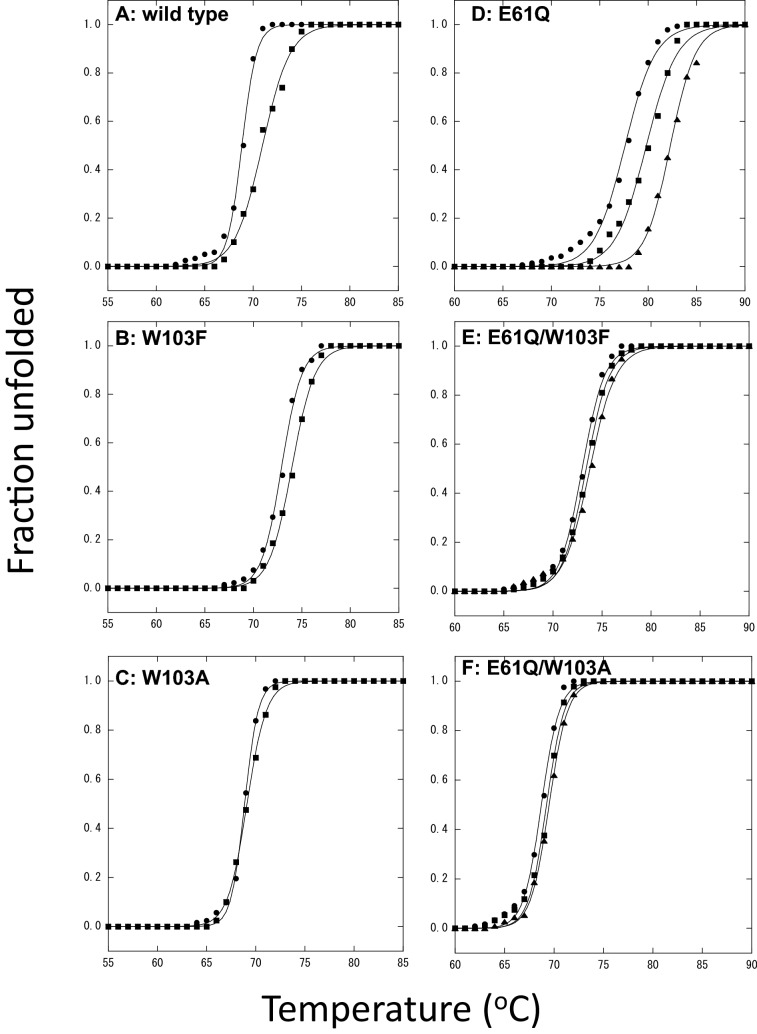
Thermal unfolding curves of wild-type **(A)**, W103F **(B)**, and W103A **(C)** in the absence (circle) or presence of (GlcNAc)_2_ (square), and those of E61Q **(D)**, E61Q/W103F **(E)**, and E61Q/W103A **(F)** in the absence (circle) or presence of (GlcNAc)_2_ (square) or (GlcNAc)_6_ (triangle). Protein solution was dialyzed against 50 mM sodium acetate buffer pH 5.0. CD value at 222 nm was monitored using a Jasco J-720 spectropolarimeter, raising the solution temperature. Final concentrations of the enzyme and (GlcNAc)_n_ were 8 μM and 8 mM, respectively.

To evaluate the binding abilities of the wild-type BcChi-A, W103F, and W103A, we determined the thermal unfolding curves of individual proteins in the absence or presence of the ligand (GlcNAc)_2_. As shown in Fig. 2A–C, the addition of the ligand significantly elevated the transition temperature of thermal unfolding (*T*_m_), and the elevations in *T*_m_ (∆*T*_m_) were 2.1 °C in the wild-type, 1.1 °C in W103F, and 0.2 °C in W103A. The mutation effects (∆∆*T*_m_) were 1.0 °C for W103F and 1.9 °C for W103A. Similar experiments were conducted using the inactive mutants, E61Q, E61Q/W103F, and E61Q/W103A, and (GlcNAc)_2_ and (GlcNAc)_6_ as the ligands. The results are shown in Fig. 2D–F. ∆*T*_m_ values upon addition of (GlcNAc)_2_ were similar to those observed when the dimer was added to the active enzymes, wild-type, W103F and W103A. The ∆∆*T*_m_ values were 1.8 °C for E61Q/W103F and 1.6 °C for E61Q/W103A). However, the higher ∆*T*_m_ values were obtained by the addition of (GlcNAc)_6_ (4.7 °C in E61Q, 0.8 °C in E61Q/W103F, and 0.8 °C in E61Q/W103A). The mutation effects (∆∆*T*_m_) were 3.9 °C for both E61Q/W103F and E61Q/W103A. The experiments were conducted two or three times to confirm the ∆∆*T*_m_ values obtained (statistical error, within ± 0.3 °C). Table 2 summarizes all *T*_m_ values obtained in the thermal unfolding experiments. Based on the ∆∆*T*_m_ values, the W103 mutations were found to significantly reduce the binding ability of BcChi-A. However, the data obtained here were not fully quantitative for evaluation of the tryptophan residue contribution to the ligand binding. For example, the differences in ∆∆*T*_m_ values observed were somewhat ambiguous between E61Q/W103F and E61Q/W103A. Thus, we tried to determine the binding ability using NMR spectroscopy.

**Table 2:** Transition temperatures of thermal unfolding (*T*_m_) for BcChi-A and the Trp103-mutated enzymes in the presence or absence of (GlcNAc)_n_.

	*T*_m_ (°C)	∆*T*_m_ (°C)	∆∆*T*_m_ (°C)
Wild-type	68.9		
Wild-type + (GlcNAc)_2_	71.0	2.1	
W103F	72.9		
W103F + (GlcNAc)_2_	74.0	1.1	1.0
W103A	68.9		
W103A + (GlcNAc)_2_	69.1	0.2	1.9
E61Q	77.6		
E61Q + (GlcNAc)_2_	79.8	2.2	
E61Q + (GlcNAc)_6_	82.3	4.7	
E61Q/W103F	73.0		
E61Q/W103F + (GlcNAc)_2_	73.4	0.4	1.8
E61Q/W103F + (GlcNAc)_6_	73.8	0.8	3.9
E61Q/W103A	68.7		
E61Q/W103A + (GlcNAc)_2_	69.3	0.6	1.6
E61Q/W103A + (GlcNAc)_6_	69.5	0.8	3.9

### Two-dimensional ^1^H-^15^N HSQC spectra of the BcChi-A mutants

**Figure 3:**
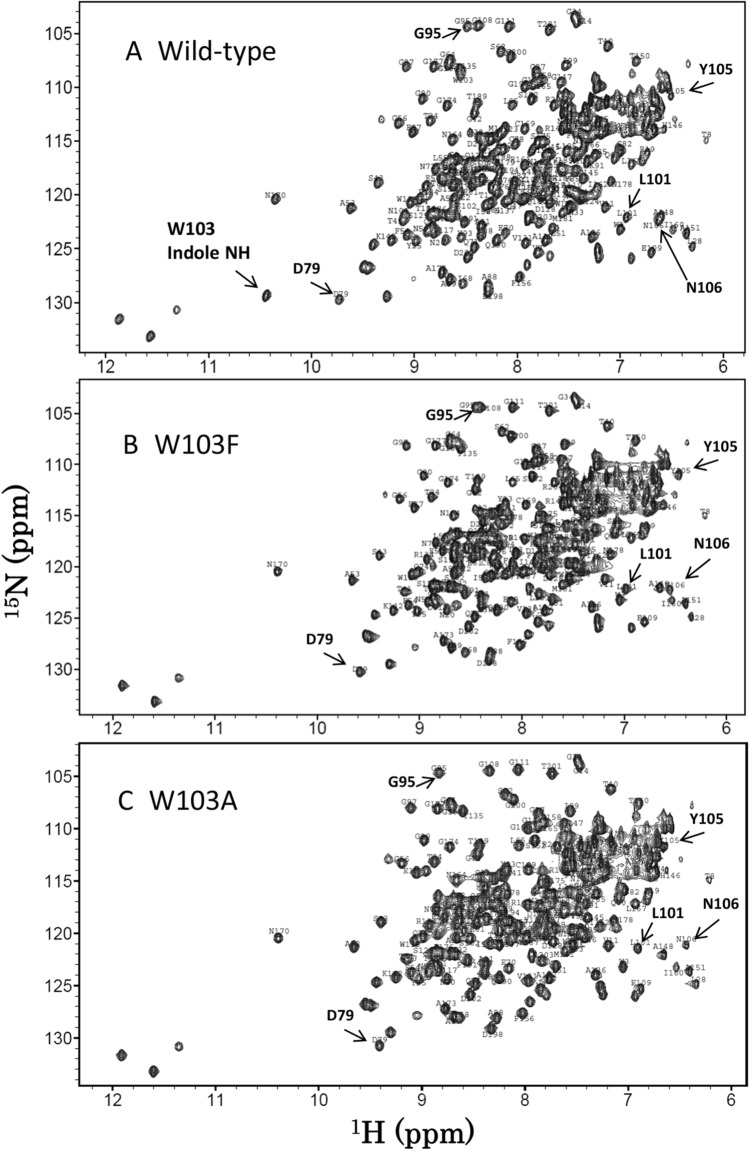

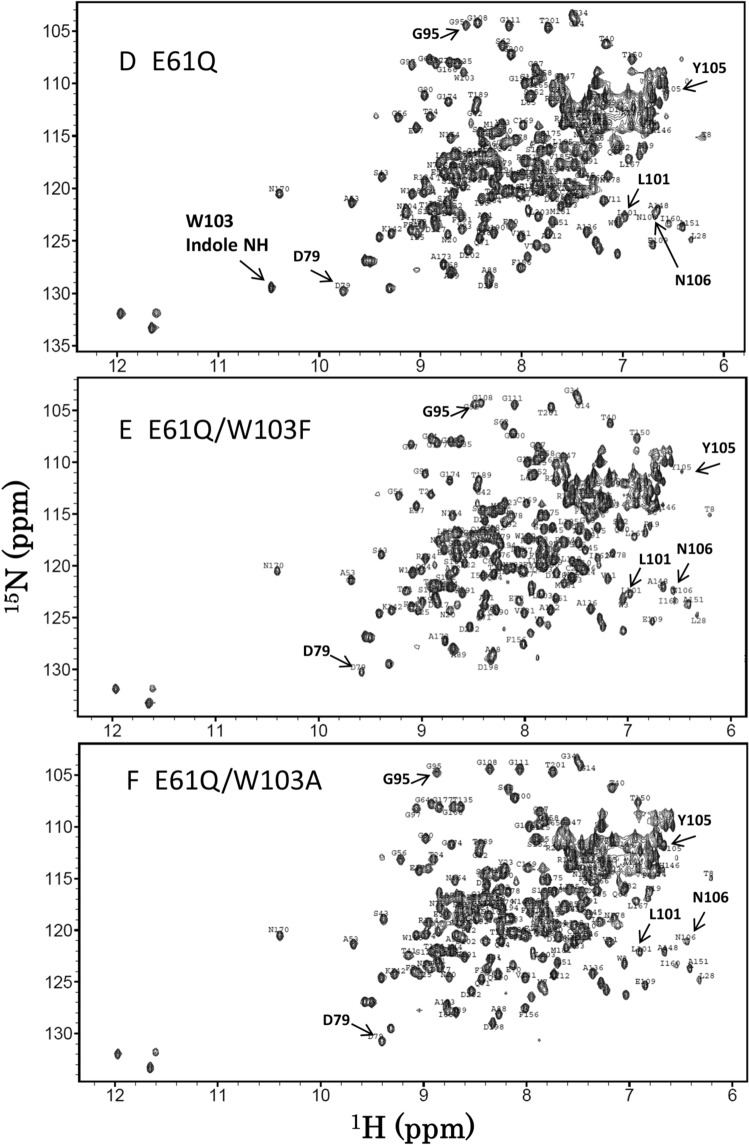
The ^1^H-^15^N HSQC spectra of wild-type **(A)**, W103F **(B)**, W103A **(C)**, E61Q **(D)**, E61Q/W103F **(E)**, and E61Q/W103A **(F)**. NMR samples contained 0.4 mM protein in 50 mM sodium acetate buffer pH 5.0 (90% H_2_O/10% D_2_O). All NMR spectra were acquired at 300 K using a Bruker AV500 spectrometer controlled with TopSpin 3.0 software. Sequential assignments were conducted referring to the assignments reported previously^36^.

Figure 3A–F show the ^1^H-^15^N HSQC spectra of the wild-type BcChi-A, W103F, W103A, E61Q, E61Q/W103F, and E61Q/W103A, respectively. Sequential assignments of the main-chain NH resonances except prolines were successfully conducted using the three-dimensional spectra, HNCACB, CBCA(CO)NH, HNCA, HNCACO, HNCO, and HNCOCA, of the individual proteins, and the assignment results are labeled nearby the individual HSQC resonances. The assignment data were deposited in Biological Magnetic Resonance Bank (BMRB, http://www.bmrb.wisc.edu/) with code numbers, 26,302 for E61Q, 26,304 for W103F, 26,303 for W103A, 26,306 for E61Q/W103F, and 26,305 for E61Q/W103A. The side-chain NH resonance of Trp103 could be assigned by comparison between the spectra as shown in the figure. In the tryptophan side-chain NH region (left-lower region of the spectra), one of the HSQC resonances observed in the spectra of the wild-type and E61Q (Fig. 3A,D) is missing in the spectra of the other four Trp103-mutated proteins (Fig. 3B,C,E,F). Thus, the missing resonance was assigned to the Trp103 side-chain NH.

**Figure 4:**
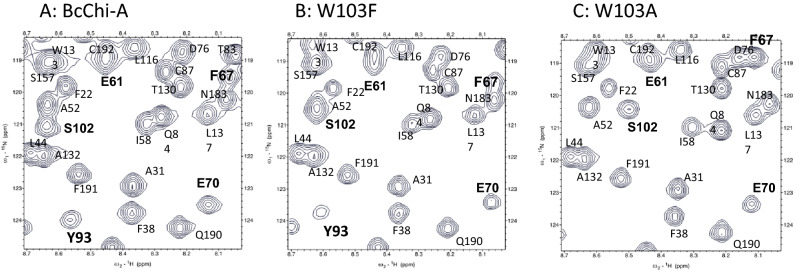
Close-up views of the central regions of the HSQC spectra of the wild-type **(A)**, W103F **(B)**, and W103A (**C**). HSQC resonances of the catalytic triad, Glu61, Glu70, and Ser102, are highlighted by bold characters. The resonances of Phe67 and Tyr93 are also highlighted because of their remarkable perturbations upon the mutations. Experimental conditions are the same as in Fig. 3.

**Figure 5:**
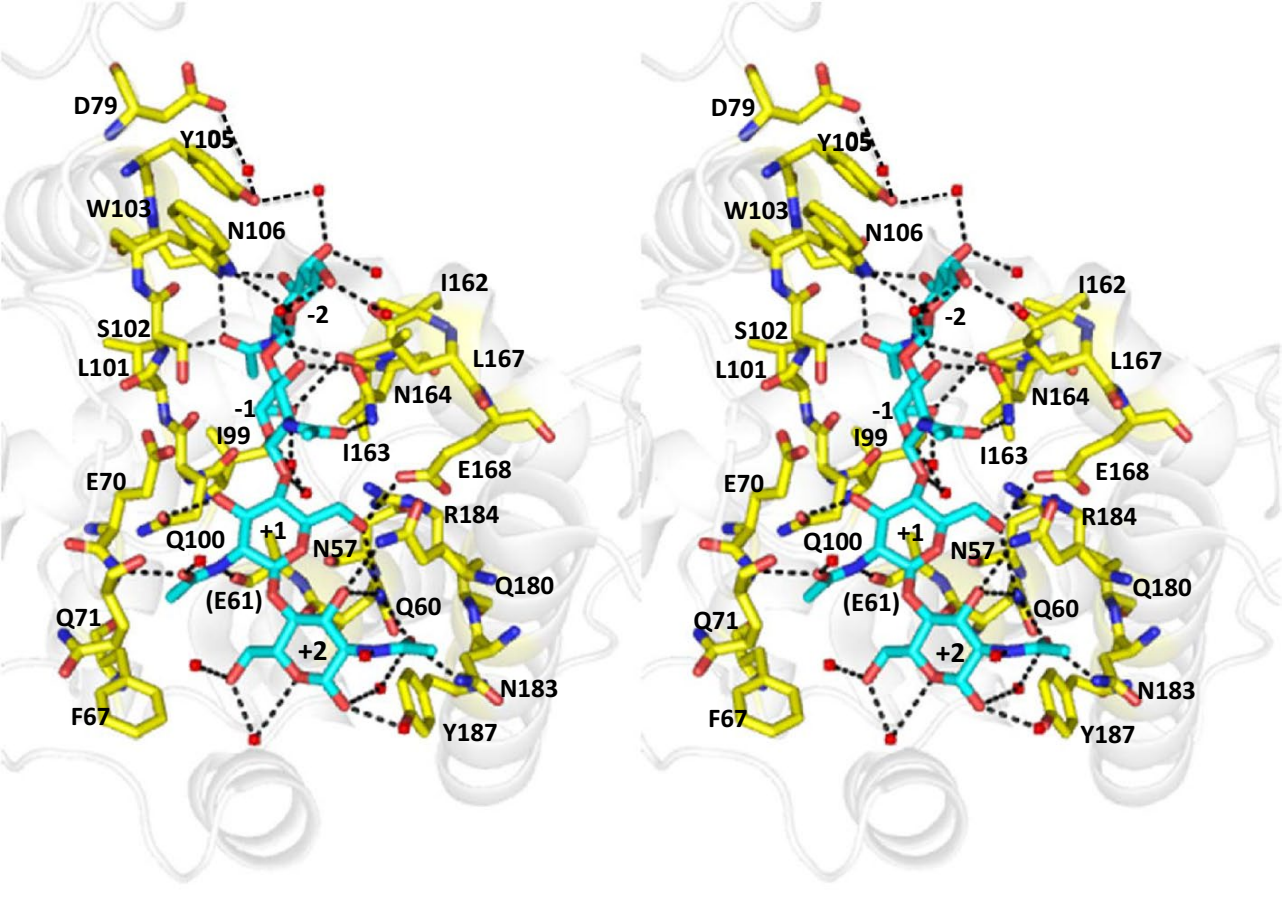
Stereo view of the substrate-binding groove of inactive mutant (E61A) BcChi-A in complex with (GlcNAc)_4_ (PDB code: 3WH1)^23^. The dotted lines represent the possible hydrogen bonds involved in sugar residue interactions. Amino acid residues, which appear to directly or indirectly involved in the interactions with (GlcNAc)_4_, are labeled. Furthermore, Phe67 and Asp79 are labeled because of their significant chemical shift perturbations upon mutation of Trp103 (Fig. 4) and upon the addition of (GlcNAc)_2_ (Fig. 6), respectively.

Judging from the overall profiles of the spectra, the E61Q mutation did not significantly affect the profile, whereas the profile was moderately affected by the W103F mutation and more intensively affected by the W103A mutation. The effect of the W103A mutation on the protein conformation appeared to be larger than that of the W103F mutation. Typical examples of the larger effects of the W103A mutation were seen in the amino acids located in the β-hairpin region, such as Gly95, Leu101, Tyr105, and Asn106 (Fig. 3). Figure 4 shows close-up views of the central region of the HSQC spectra of wild-type, W103F, and W103A. Although the HSQC resonances of catalytic residues, Glu61 and Glu70, were not significantly affected by the Trp103 mutations, the larger effects were observed in the resonances of Ser102 and Phe67; the former is the nearest neighbor of the mutated amino acid Trp103, and the latter is located at the bottom of catalytic cleft, as revealed by the crystal structure shown in Fig. 5. The larger effect of the W103A mutation was also observed in the resonance of Tyr93 (Fig. 4); however, the side-chain moiety of Tyr93 is oriented away from the bound sugar in the crystal structure (Fig. 5), and unlikely involved in the sugar residue binding. In any case, the W103A mutation appeared to widely affect the protein conformation from the glycon-binding site (negatively-numbered subsites) to the catalytic cleft. Closer examination of the spectra of W103F and E61Q/W103F revealed that the W103F mutation resulted in small but significant changes in the chemical shifts of Asp79, Gly95, Tyr105 and Asn106 (Fig. 3), which are located nearby Trp103 in the crystal structure (Fig. 5).

### Effects of the addition of (GlcNAc)_n_ on the spectra

**Figure 6:**
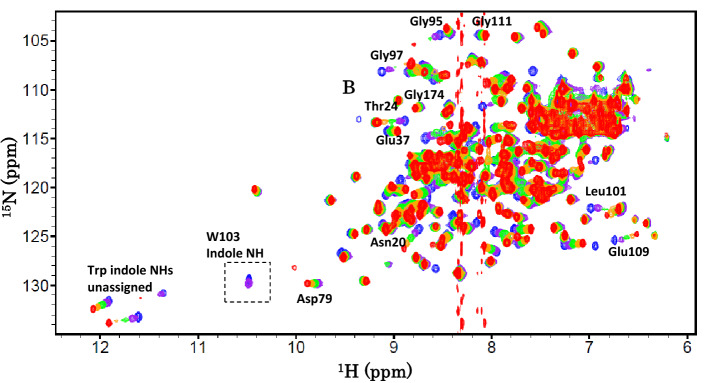
Overlaid HSQC spectra of wild-type BcChi-A in the absence or presence of increasing concentrations of (GlcNAc)_2_. The protein solution (0.2 mM) was prepared in 50 mM sodium acetate buffer pH 5.0. Concentrations of (GlcNAc)_2_ added were 0 mM (blue), 2 mM (purple), 5 mM (green), 10 mM (orange), and 50 mM (red). The spectra were recorded as in Fig. 3. The amino acids whose resonances clearly responded (Dd > 0.1 ppm) to the addition of (GlcNAc)_2_ are labeled in the spectra.

Many HSQC resonances of wild type BcChi-A were affected by the addition of (GlcNAc)_2_, as shown in Fig. 6. The resonances labeled in the spectrum gradually shifted with progress of the (GlcNAc)_2_ titration, indicating a fast exchange rate between the free and bound states. The amino acid residues whose resonances shifted upon the addition of (GlcNAc)_2_ were similar to those reported previously for E61A BcChi-A mutant^20^, and distributed in the entire region of the substrate-binding groove. Most resonances affected showed similar relative shifts in response to increasing ligand concentrations, indicating that the domain motion induced by the ligand binding cooperatively took place^19^. No significant changes were observed in amino acids showing the shifts upon the addition of (GlcNAc)_2_, when the Trp103-mutated enzymes were used instead of wild type.

It should be noted that the side-chain NH resonance of Trp103 was gradually broadened, and became finally beyond recognition upon the addition of (GlcNAc)_2_, suggesting a hydrogen bond formation of the side-chain NH of Trp103.

### Binding affinities of (GlcNAc)_n_

**Figure 7:**
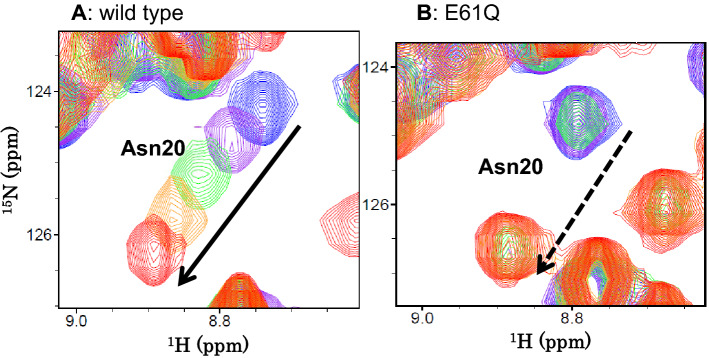
**(A)** Overlaid HSQC resonance of Asn20 of wild-type BcChi-A upon addition of increasing concentration of (GlcNAc)_2_. Experimental conditions were the same as in Fig. 6. **(B)** Overlaid Asn20 resonance of E61Q upon addition of (GlcNAc)_6_. The E61Q solution (0.2 mM) was prepared in 50 mM sodium acetate buffer pH 5.0. Concentrations of (GlcNAc)_6_ added were 0 mM (blue), 0.04 mM (purple), 0.08 mM (green), 0.16 mM (orange), and 0.8 mM (red). The spectra were recorded as in Fig. 3.

**Figure 8:**
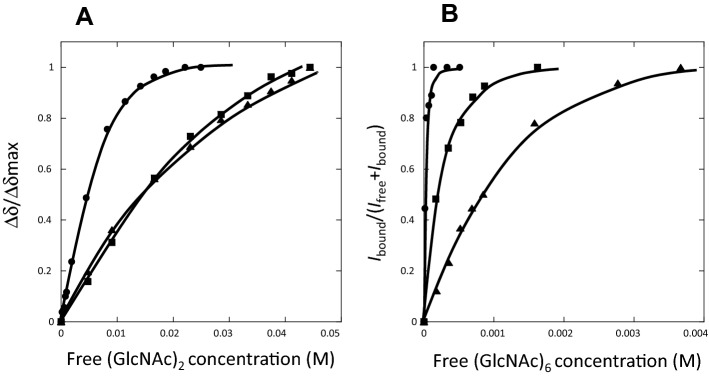
Titration curves of (GlcNAc)_n_ (n = 2 or 6) binding to BcChi-A proteins as determined by chemical shift perturbations or intensity changes of the Asn20 resonance. **(A)** (GlcNAc)_2_ titration to wild-type (circle), W103F (triangle), and W103A (square). **(B)** (GlcNAc)_6_ titration to E61Q (circle), E61Q/W103F (triangle), and E61Q/W103A (square). Experimental conditions were the same as in Figs. 6 and 7. Nonlinear least fitting analysis of the titration curves provided the *K*_d_ and ∆*G*° values as listed in Table 3.

To quantitatively determine the binding affinities of wild-type BcChi-A and the mutants, we selected the main-chain NH resonance of Asn20, which was clearly separated from the other resonances. Furthermore, Asn20 is localized to the hinge between the two domains, which is most strongly affected by the domain motion induced by (GlcNAc)_n_ binding^19,20^. Thus, Asn20 was regarded as most appropriate for monitoring the changes in chemical shift or intensity. No significant difference was observed between the binding constants obtained from the chemical shift perturbations of different HSQC resonances of the BcChi-A enzymes shown in Fig. 6, because the domain motion cooperatively took place upon the (GlcNAc)_n_ binding. As shown in Fig. 7A, the HSQC resonance migrated continuously with the increase in (GlcNAc)_2_ concentration, and the chemical shift changes were measured accurately at individual (GlcNAc)_2_ concentrations. The relative chemical shift changes (Δδ) were calculated according to Eq. (1) and plotted against the free (GlcNAc)_2_ concentrations to obtain the titration curves for wild-type, W103F, and W103A. As shown in Fig. 8A, the chemical shift change of the Asn20 resonance was less intense in W103F and W103A than in wild-type. The binding constants were obtained using a non-linear least square fitting procedure based on Eq. (2) and are listed in Table 3 together with their binding free energy changes. The W103 mutations were found to reduce the binding affinity by about 1.0 kcal/mol.

**Table 3:** Binding affinity calculated from the chemical shift perturbation of Asn20 upon the addition of (GlcNAc)_n_.

Chitinase	Ligand	*K*_d_ (M)	*K*_assoc_ (M^−1^)	∆*G*° (kcal/mol)
Wild-type	(GlcNAc)_2_	4.2 × 10^–3^	2.4 × 10^2^	− 3.2
W103F	(GlcNAc)_2_	2.2 × 10^–2^	2.6 × 10	− 2.3
W103A	(GlcNAc)_2_	2.4 × 10^–2^	2.1 × 10	− 2.2
E61Q	(GlcNAc)_6_	1.4 × 10^–5^	7.3 × 10^4^	− 6.7
E61Q/W103F	(GlcNAc)_6_	1.0 × 10^–3^	1.0 × 10^3^	− 4.1
E61Q/W103A	(GlcNAc)_6_	0.2 × 10^–3^	5.0 × 10^3^	− 5.0

When (GlcNAc)_6_ was added to the inactive mutant E61Q solution, the resonance intensity of Asn20 decreased gradually without changing the chemical shift, and the resonance gradually became visible at the migration position (Fig. 7B), indicating a slow exchange rate between the free and bound states. This suggested that (GlcNAc)_6_ may bind to the inactive mutant enzyme with higher affinity. In this case, we measured the integral (volume) of the Asn20 resonance for the individual (GlcNAc)_6_ concentrations, and the relative increases in the integral of Asn20 resonance at bound state were plotted against the free (GlcNAc)_6_ concentrations (Fig. 8B) to obtain the titration curves for E61Q, E61Q/W103F, and E61Q/W103A. The binding constants and the free energy changes of binding were similarly obtained, and are listed in Table 3. The W103 mutations were found to strongly reduce the binding affinity by 1.7–2.6 kcal/mol.

## Discussion

Mutations of Trp103 to phenylalanine and alanine in BcChi-A significantly reduced the enzymatic activities toward glycol chitin and chitooligosaccharide substrates (Table 1). To tentatively identify the role of Trp103 in the enzymatic reaction, we tried to examine the binding abilities of W103F and W103A using thermal unfolding experiments. BcChi-A does not hydrolyze (GlcNAc)_2_ but does (GlcNAc)_n_ with a polymerization degree of more than 3^33^. Thus, we used the inactive mutants (E61Q. E61Q/W103F, and E61Q/W103A) for analyzing the binding ability of (GlcNAc)_6_, while (GlcNAc)_2_-binding abilities were evaluated using active ones (wild-type, W103F, and W103A). From the ∆*T*_m_ values listed in Table 2, (GlcNAc)_6_ was found to more strongly bind to the enzymes. The greater ∆*T*_m_ values in wild-type and E61Q compared with those obtained for Trp103-mutated enzymes clearly showed importance of Trp103 in the (GlcNAc)_n_ binding (Fig. 2 and Table 2). In our previous report, chitooligosaccharide binding to BcChi-A was investigated by isothermal titration calorimetry (ITC), which provided the reliable values of thermodynamic parameters for the binding interactions^33^. Thus, using ITC, we tried to thermodynamically determine the binding abilities of Trp103-mutated BcChi-A to evaluate the free energy contribution of Trp103 to the interaction with the substrate. However, the ITC measurements of Trp103-mutated enzymes were unsuccessful, because no heat release/absorption was observed upon titrations of (GlcNAc)_n_ to W103F and W103A. NMR spectroscopy is one of the most reliable methods for quantitative determination of the protein–ligand interaction^34,35^. Sequential assignment of the HSQC resonances of BcChi-A was carried out in our laboratory and reported previously^36^, facilitating NMR investigation of the BcChi-A enzyme. Here, we analyzed the (GlcNAc)_n_-binding interactions of BcChi-A based on the HSQC spectra of the wild-type and Trp-mutated enzymes.

### Mutational effect on the binding affinity

(GlcNAc)_2_ titration to wild-type BcChi-A resulted in significant line-broadening of the side-chain NH resonance of Trp103, which became finally beyond recognition (Fig. 6). The line-broadening of the Trp103 side-chain NH resonance was similarly observed, when (GlcNAc)_6_ was titrated into the E61Q solution. These results suggested hydrogen bond formation of the Trp103 side-chain NH. In fact, the crystal structure of BcChi-A in complex with (GlcNAc)_4_ revealed a possible hydrogen bond between the side-chain NH of Trp103 and the oxygen atom of the pyranose ring of -2 GlcNAc (Fig. 5). The NMR titration experiments (Figs. 7 and 8) successfully provided the binding affinities and revealed again the importance of Trp103 in the sugar residue binding, as listed in Table 3. The results are fully consistent with the data obtained from the thermal unfolding experiments (Table 2). However, the (GlcNAc)_2_ binding affinity to W103F (− 2.3 kcal/mol) was similar to that to W103A (− 2.2 kcal/mol), whereas the (GlcNAc)_6_ affinity to E61Q/W103F (− 4.1 kcal/mol) was significantly lower than that to E61Q/W103A (− 5.0 kcal/mol). The discrepancy between the two sets of the binding affinities (Table 3; the upper three lines and the lower three lines) may result from the smaller contact area of the bound (GlcNAc)_2_. (GlcNAc)_2_ bound to the substrate-binding groove of BcChi-A did not always make a contact with the 103rd amino acid, but (GlcNAc)_6_ bound to the inactive mutants of BcChi-A was mostly in contact with the 103rd amino acid. The data set obtained from the (GlcNAc)_6_ titration to the inactive mutants are likely to more correctly reflect the mutation effects.

### Effect on the amino-acid network involved in GlcNAc binding

Mutation effects of Trp → Ala are usually more extensive than those of Trp → Phe, because the hydrophobic phenyl group is retained after Trp → Phe but not after Trp → Ala. This idea is consistent with the NMR spectra, in which the chemical shift perturbations were more extensive in W103A than in W103F (Figs. 3 and 4). However, the effects of tryptophan mutations on the binding affinities obtained in this study (Table 3) were unusual. Mutation effects were larger in E61Q/W103F than in E61Q/W103A. To rationalize the unusual effects on the binding affinities, we tried to obtain the crystals of W103F and W103A, but the trials were unsuccessful. Thus, we closely examined the NMR spectra of the BcChi-A enzymes (Figs. 3 and 4). As described in “Results”, chemical shift perturbations induced by the W103F mutation were observed in Asp79, Tyr105, and Asn106. The latter two amino acids and Trp103 form a hydrogen-bonding network with the -2 GlcNAc as shown in the crystal structure (Fig. 5), and they are highly conserved (conservation grades 7–9) in “loopless” GH19 enzymes except the bacterial ones (Fig. 1 and Supplementary Fig. S1). This indicated that the W103F mutation not only eliminated the hydrogen bond formation by the tryptophan side-chain NH but also affected the sugar residue interaction of Tyr105 and Asn106. As shown in Fig. 5, the Trp103 aromatic ring appears to partly stack with the Tyr105 ring. The W103F mutation eliminated the hydrogen bond with the -2 sugar, and then the Phe103 ring may have more strongly stacked with the Tyr105 ring, reducing the binding ability of Tyr105. Furthermore, the increased hydrophobicity around Phe103 may have affected the polarity of Asn106. These effects may have strongly reduced the binding affinity toward (GlcNAc)_6_ (6.7 → 4.1 kcal/mol). However, in the W103A mutation, Ala103 may not have such effects on Tyr105 and Asn106. The reduction in binding affinity (6.7 → 5.0 kcal/mol) may be only derived from missing the hydrogen bond with the -2 sugar and from the lower hydrophobicity of Ala103.

### Effect on the catalytic cleft

Enzymatic activities reduced by Trp103 mutations (100%, 40/58%, and 13/19% for BcChi-A, W103F, and W103A, respectively) were not consistent with the decreases in binding affinity (Tables 1 and 3). The lowest activity in W103A suggested that the W103A mutation also affected the catalytic potency. Unfortunately, kinetic analysis of the W103F and E103A was not successful, because of their strong substrate inhibition. Consequently, we could not obtain the catalytic turnover number (*k*_cat_). Since crystallization studies on W103F and W103A were also unsuccessful, we repeated a close examination of the HSQC spectra of the W103F and W103A (Fig. 4) paying attention to the catalytic triad, Glu61, Glu70 and Ser102, which is the nearest neighbor of the mutation site Trp103. In the catalytic reaction, the glycosidic linkage between -1 GlcNAc and + 1 GlcNAc is first split off by the action of Glu61, then the water molecule activated by conjugate base of the side chain of Glu70 attacks the C1 carbon of -1 GlcNAc to complete hydrolysis^20^. The water molecule involved in hydrolysis is positioned by the side-chain hydroxyl of Ser102. As seen from Fig. 4, neither W103F nor W103A mutations resulted in significant changes in the HSQC resonances of Glu61 and Glu70, whereas the HSQC resonance of Ser102 was strongly perturbed in W103A but weakly in W103F. This clearly showed that the W103A mutation brought about the significant changes in the environment of Ser102 but the W103F mutation did not. The hydrophobicities of tryptophan and phenylalanine are higher than that of alanine. This situation may cause significant changes in the state of the catalytic cleft. This was confirmed from the chemical shift perturbation of the resonance of Phe67 (Fig. 4C), which is located in the bottom portion of the catalytic cleft, as shown in the crystal structure (Fig. 5). This may have resulted in the intense reduction in catalytic potency, and hence the lowest enzymatic activity of W103A (13%). Because the effect of the W103F mutation on the catalytic cleft was negligible, the decrease in the enzymatic activity in W103F (40/58%) may mainly result from the lowest binding ability. Thus, we concluded that Trp103 does not only bind the -2 GlcNAc but also controls the states of other amino acids responsible for substrate binding (Leu101, Tyr105, and Asn106) and catalytic reaction (Ser102).

### Tryptophan residues in the lysozyme superfamily

As shown in Fig. 1A, Trp103 is highly conserved in the “loopless” GH19 chitinases (Fig. 1 and Supplementary Fig. S1), but not in the “loopful” chitinases. In divergent evolution of the lysozyme superfamily, a multi-functional tryptophan may have been selected as the 103rd amino acid of the “loopless” GH19 chitinases to compensate for the loss of binding affinity derived from lacking the loop structures, that are responsible for substrate binding. This idea can be applied to the conserved tryptophan residue (Trp62) in GH22 lysozymes, whose binding grooves are even shorter than those of “loopless” GH19 chitinases (Fig. 1B). It should be noted that an additional tryptophan residue (Trp63) has been selected and conserved to interact with -2 GlcNAc in the GH22 lysozymes. In the shortest binding groove of GH22 lysozymes, the contiguous tryptophan residues (Trp62/Trp63) appear to act towards the sugar residues from the solvent-exposed and the hydrophobic sides, respectively (Fig. 1B). Trp62/Trp63 in GH22 lysozymes may be more advantageous for accommodating the peptidoglycan chains consisting of alternating two amino sugars, MurNAc and GlcNAc. In the crystal structure of the lysozyme-(GlcNAc)_n_ complex, Trp62 contributes to the substrate binding not only with the hydrogen bond toward -2 GlcNAc but also with CH-π stacking toward -3 GlcNAc^37^. Kuhara et al.^38^ estimated the free energy contribution of the individual subsites of hen egg white lysozyme and its Trp62-modified lysozymes. They reported that effects of the Trp62 modification extended from the -2 site to the -3 and -4 sites. Another mutational study on Trp62 of lysozyme revealed that the Trp62 mutation did not significantly change the catalytic turnover number^37^. Thus, Trp62 of lysozymes contributes to the binding interactions with the GlcNAc residues bound to subsites -2, -3, and -4, but not to the catalytic action. Although the relative location of Trp103 is apparently similar to that of Trp62 in lysozymes (Fig. 1B), the multi-functional Trp103 in GH19 chitinases appears to contribute to the enzymatic reaction in a manner different from that of the multi-functional Trp62 in the GH22 lysozymes. The difference may be due to shifting of Trp103 (GH19) from Trp62 (GH22) by two amino acid units in the sequence alignment (Fig. 1A). In any case, there is no doubt that both tryptophan residues with such a multi-functionality are selected and conserved in the invariant β-hairpin region of enzymes with shorter binding grooves in the divergent evolution of the lysozyme superfamily^24,25^.

## Methods

### Materials

Chitooligosaccharides, (GlcNAc)_n_ (n = 2–6), were obtained by acid hydrolysis of chitin^39^, and purified by gel filtration on Cellufine Gcl-25 m (JNC Co., Tokyo, 3.5 × 180 cm). Stable isotope-labeled compounds (D_2_O, ^15^NH_4_Cl, and ^13^C-glucose) were the products of Cambridge Isotope Lab. *Escherichia coli* BL21(DE3) cells were purchased from Novagen (Madison, WI). Q Sepharose and Sephacryl S-100 h were from GE Healthcare (Tokyo, Japan). Other reagents were of analytical grade commercially available.

### Site-directed mutagenesis

Mutations of Trp103 of BcChi-A to phenylalanine or alanine (W103F or W103A) were done using a QuikChange site-directed mutagenesis kit (Stratagene). The oligonucleotide primers used were 5′-CCAATCCAACTCTC ATTTAACTACAACTATG-3′ (W103F) and 5′-CCAATCCAACTCTCAGCGAAC TACAACTATG-3′ (W103A), where the underlined regions are mutation sites. These mutations were introduced into the cDNA fragments of wild-type BcChi-A and E61Q, that had been obtained previously^33^, to obtain the single mutants (W103F and W103A) and the double mutants (E61Q/W103F and E61Q/W103A), respectively. The mutated cDNA fragments were then sequenced to verify the presence of the desired mutation. The mutant fragments were recovered and ligated into the expression vector pET-22b.

### Protein expression and purification

The recombinant proteins, wild-type BcChi-A, W103F, W103A, E61Q, E61Q/W103F, and E61Q/W103A, were obtained using the methods previously described^20,33,40^. Briefly, the wild-type and mutated plasmids, pET-BcChi-A, pET-BcChi-A-W103F, pET-BcChi-A-W103A, pET-BcChi-A-E61Q, pET-BcChi-A-E61Q/W103F, and pET-BcChi-A-E61Q/W103A, were respectively introduced into *Escherichia coli* BL21(DE3). *E. coli* cells harboring the plasmid were grown to OD_600 nm_ = 0.6 before induction with 1 mM isopropyl thiogalactoside. After cultivation for 24 h at 18 °C, cells were disrupted in 20 mM Tris–HCl buffer (pH 7.5) with a sonicator. The supernatant fraction obtained after the acid treatment (pH 4.0) was dialyzed against 10 mM sodium acetate buffer (pH 5.0) and applied to Q-Sepharose Fast Flow column chromatography, followed by gel-filtration on Sephacryl S-100 h. The purity of the enzyme preparation was confirmed by SDS-PAGE in accordance with the method of Laemmli^41^. Protein concentrations were determined by reading the absorbance at 280 nm, using the extinction coefficients of BcChi-A (49,390 M^−1^ cm^−1^) and its mutants obtained from the equation proposed by Pace et al.^42^.

### Chitinase activity

Chitinase activity was determined colorimetrically using glycol chitin, which was synthesized by the method reported by Yamada and Imoto^43^, as a substrate. Ten microliters of the enzyme solution was added to 500 μl of 0.2% (w/v) glycol chitin solution in 0.1 M sodium acetate buffer, pH 5.0. After incubation of the reaction mixture at 37 °C for 15 min, the reducing sugars were determined with ferri-ferrocyanide reagent using the method of Imoto and Yagishita^44^. An increase in reducing sugars was regarded as chitinase activity. One unit (U) of enzyme activity was defined as the amount of enzyme (mg) releasing 1 μmole of GlcNAc per min at 37 °C.

### HPLC analysis of enzymatic products

The reaction products from the chitinase-catalyzed hydrolysis of (GlcNAc)_5_ were quantitatively determined by gel filtration HPLC^45^. The enzymatic reaction was performed in 50 mM sodium acetate buffer, pH 5.0, at 40 °C. Enzyme concentrations were 0.041 μM for wild-type, 0.041 μM for W103F, and 0.41 μM for W103A. Substrate concentration was 4.75 mM respectively. To completely terminate the enzymatic reaction at a given incubation time, a portion of the reaction mixture was mixed with an equal volume of 0.1 M NaOH solution, and immediately frozen in liquid nitrogen. The resultant solution was applied to a gel filtration column of TSK-GEL G2000PW (Tosoh, Tokyo) and eluted with distilled water at a flow rate of 0.3 ml/min. GlcNAc and (GlcNAc)_n_ (n = 2–6) were monitored by ultraviolet absorption at 220 nm. Peak areas obtained for GlcNAc sugars were converted to molar concentrations, which were then plotted against reaction time to obtain the rate of substrate degradation (μmoles min^−1^), which was then used for calculating a specific activity (μmoles min^−1^ mg^−1^).

### Thermal unfolding experiments

To obtain the thermal unfolding curve of the protein, the CD value at 222 nm was monitored using a Jasco J-720 spectropolarimeter (cell length 0.1 cm), while the solution temperature was raised at a rate of 1 °C/min using a temperature controller (PTC-423L, Jasco)^46^. The fraction of unfolded protein at each temperature was calculated from the CD value by linearly extrapolating the pre- and post-transition baselines into the transition zone, and plotted against the temperature to obtain the normalized unfolding curves. The transition temperature of thermal unfolding (*T*_m_) was calculated based on the unfolding curves by non-linear curve fitting procedure. To evaluate the binding abilities of wild-type BcChi-A and its mutants, (GlcNAc)_n_ (n = 2 or 6) was added to the enzyme solution, and the increase in *T*_m_ (∆*T*_m_) was obtained to evaluate the binding ability for each (GlcNAc)_n_. The greater the ∆*T*_m_ value, the higher the binding affinity^46^. Final concentrations of the enzyme and (GlcNAc)_n_ were 8 μM and 8 mM, respectively.

### Stable isotope-labeling of the BcChi-A proteins

Individual recombinant expression plasmids for wild-type BcChi-A and its mutants were introduced into *E. coli* BL21(DE3). The cells harboring the plasmid were grown in M9 medium containing ^15^N-NH_4_Cl to produce ^15^N-labeled BcChi-A proteins^20,36^. ^13^C-glucose was also added to medium, producing the ^15^N/^13^C-double-labeled BcChi-A proteins. Cultivation was conducted to obtain an OD_600nm_ of 0.6 before induction with 1 M isopropyl thiogalactoside. Growth was then continued for 18 h at 25 °C. The stable isotope-labeled proteins were extracted and purified as described above.

### Sequential assignments of the BcChi-A proteins

Each BcChi-A protein solution (0.4 mM) in 50 mM sodium acetate buffer pH 5.0 (90% H_2_O/10% D_2_O) was used for NMR experiments. All NMR spectra were acquired at 300 K using a Bruker AV500 spectrometer controlled with TopSpin 3.0 software and equipped with a triple-resonance pulsed-field-gradient cryoprobe head. ^1^H chemical shifts were referenced to HDO (4.64 ppm at 30 °C) relative to trimethylsilyl propanoic acid. ^15^N and ^13^C chemical shifts were indirectly calibrated from each gyromagnetic ratio^47^. Sequential assignments were performed using ^15^N/^13^C-double-labeled BcChi-A from two-dimensional ^1^H-^15^N HSQC experiments and from three-dimensional HNCACB, CBCA(CO)NH, HNCA, HNCACO, HNCO, and HNCOCA experiments^36,48^. All spectra were processed using NMRPipe software^49^ and analyzed using Sparky software^50^.

### Ligand-binding experiments

Two-dimensional ^1^H-^15^N HSQC spectra of wild-type, W103F, and W103A were recorded for 0.2 mM ^15^N-labeled proteins in 50 mM sodium acetate buffer pH 5.0 (90%H_2_O/10%D_2_O), in the absence or presence of increasing concentrations of (GlcNAc)_2_. Chemical shift changes induced by (GlcNAc)_2_ binding (∆δ) (Fig. 7A) were calculated using the equation,

1 $$\Delta \delta = \left\{ {{{\left( {\Delta {\text{NH}}^{2} + {{\Delta {\text{N}}^{2} } \mathord{\left/ {\vphantom {{\Delta {\text{N}}^{2} } {25}}} \right. \kern-\nulldelimiterspace} {25}}} \right)} \mathord{\left/ {\vphantom {{\left( {\Delta {\text{NH}}^{2} + {{\Delta {\text{N}}^{2} } \mathord{\left/ {\vphantom {{\Delta {\text{N}}^{2} } {25}}} \right. \kern-\nulldelimiterspace} {25}}} \right)} 2}} \right. \kern-\nulldelimiterspace} 2}} \right\}^{1/2}$$

where ∆NH and ∆N represent the observed shifts in the ^1^H-axis and ^15^N-axis, respectively. The relative values of ∆δ (∆δ/∆δ_max_) were plotted against the free oligosaccharide concentrations, and the association constant, *K*_assoc_ (1/*K*_d_), was estimated by nonlinear curve fitting based on the equation,

2 $${{\Delta \delta } \mathord{\left/ {\vphantom {{\Delta \delta } {\Delta \delta_{\max } = {{[{\text{S}}]_{{{\text{free}}}} } \mathord{\left/ {\vphantom {{[{\text{S}}]_{{{\text{free}}}} } {\left( {[{\text{S}}]_{{{\text{free}}}} + {1 \mathord{\left/ {\vphantom {1 {K_{{{\text{assoc}}}} }}} \right. \kern-\nulldelimiterspace} {K_{{{\text{assoc}}}} }}} \right)}}} \right. \kern-\nulldelimiterspace} {\left( {[{\text{S}}]_{{{\text{free}}}} + {1 \mathord{\left/ {\vphantom {1 {K_{{{\text{assoc}}}} }}} \right. \kern-\nulldelimiterspace} {K_{{{\text{assoc}}}} }}} \right)}}}}} \right. \kern-\nulldelimiterspace} {\Delta \delta_{\max } = {{[{\text{S}}]_{{{\text{free}}}} } \mathord{\left/ {\vphantom {{[{\text{S}}]_{{{\text{free}}}} } {\left( {[{\text{S}}]_{{{\text{free}}}} + {1 \mathord{\left/ {\vphantom {1 {K_{{{\text{assoc}}}} }}} \right. \kern-\nulldelimiterspace} {K_{{{\text{assoc}}}} }}} \right)}}} \right. \kern-\nulldelimiterspace} {\left( {[{\text{S}}]_{{{\text{free}}}} + {1 \mathord{\left/ {\vphantom {1 {K_{{{\text{assoc}}}} }}} \right. \kern-\nulldelimiterspace} {K_{{{\text{assoc}}}} }}} \right)}}}}$$

The free oligosaccharide concentrations [S]_free_ were obtained by subtracting the bound oligosaccharide concentration [ES] from the total oligosaccharide concentration [S]_total_. Similarly, the spectra of E61Q, E61Q/W103F, and E61Q/W103A were recorded in the absence or presence of increasing concentrations of (GlcNAc)_6_. Since the interaction of (GlcNAc)_6_ was a slow exchange (Fig. 7B), ∆δ values could not be determined for individual titration points. Thus, the relative intensities (integral) of the bound state to the total intensity of the free and bound states (*I*_bound_/(*I*_free_ + *I*_bound_)) were plotted instead of ∆δ/∆δ_max_ to obtain the association constant, *K*_assoc_ (1/*K*_d_).

## Supplementary Information

Supplementary Information.

## Abbreviations

GH: Glycosyl hydrolase
BcChi-A: A “loopless” GH19 chitinase from *Bryum coronatum*
E61Q, W103F, andW103A: BcChi-A single mutants, in which Glu61 or Trp103 is mutated to the corresponding amino acids
E61Q/W103F andE61Q/W103A: BcChi-A double mutants having the two mutations at Glu61 and Trp103, GlcNAc, *N*-acetylglucosamine
(GlcNAc)_n_: β-1,4-Linked oligosaccharides of GlcNAc with a polymerization degree of n
NMR: Nuclear magnetic resonance
HSQC: Heteronuclear single-quantum coherence spectroscopy
CD: Circular dichroism
