# Supplementary material for: Multi-functionality of a tryptophan residue conserved in substrate-binding groove of GH19 chitinases
Source: Sci Rep. 2021 Jan 28;11:2494. doi: 10.1038/s41598-021-81903-3 (PMC7844276; doi:10.1038/s41598-021-81903-3)

Multi-functionality of a tryptophan residue conserved in substrate-binding groove of GH19 chitinases

Takuya Nagata, Shoko Shinya, Takayuki Ohnuma, and Tamo Fukamizo

Supplementary Fig. 1

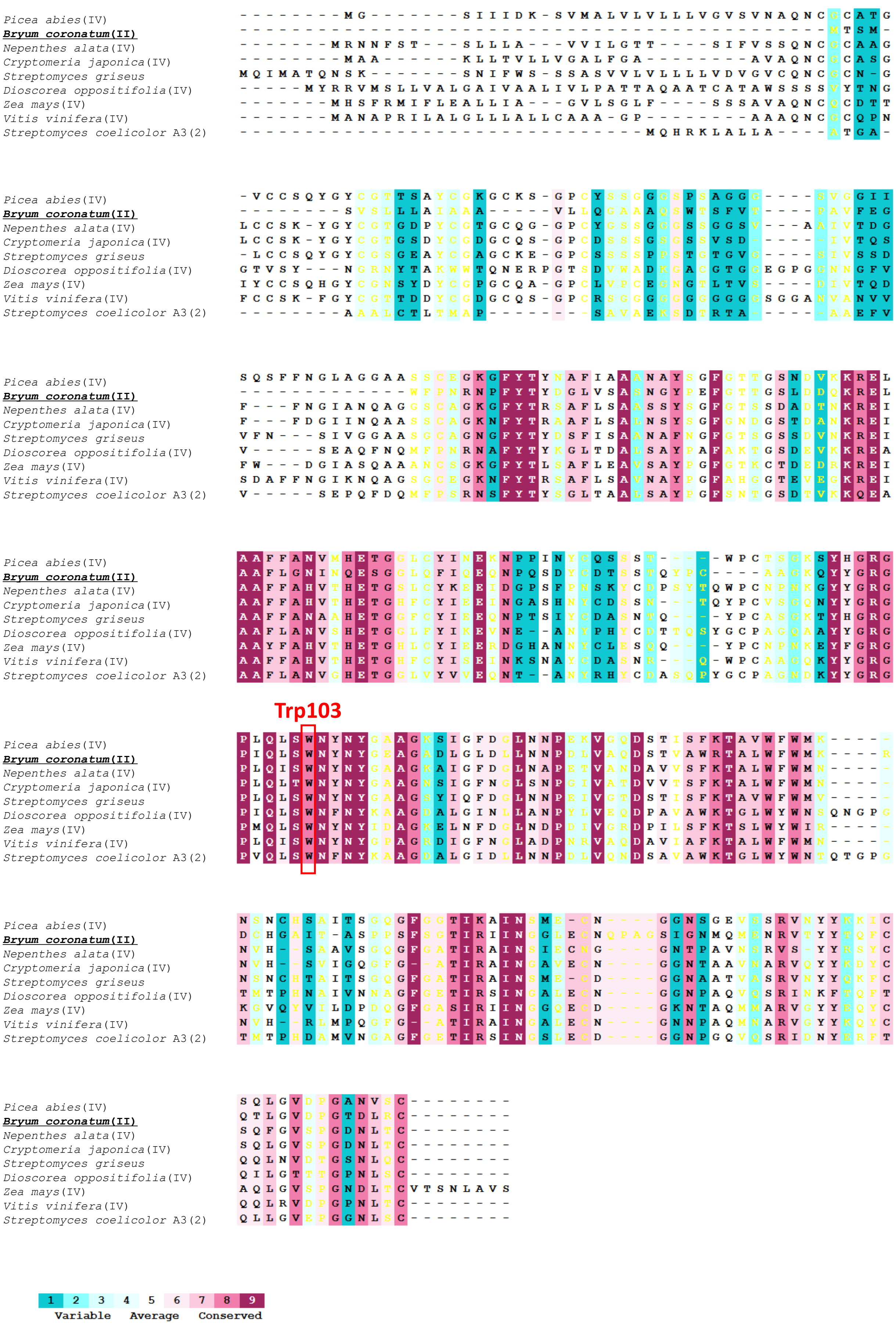

Supplement: Supplementary file 1 — Supplementary Information. [file 41598_2021_81903_MOESM1_ESM.pdf]
